# Quantification of bioluminescence from the surface to the deep sea demonstrates its predominance as an ecological trait

**DOI:** 10.1038/srep45750

**Published:** 2017-04-04

**Authors:** Séverine Martini, Steven H. D. Haddock

**Affiliations:** 1 Monterey Bay Aquarium Research Institute (MBARI), 7700 Sandholdt Road, Moss Landing, 95039, CA, USA

## Abstract

The capability of animals to emit light, called bioluminescence, is considered to be a major factor in ecological interactions. Because it occurs across diverse taxa, measurements of bioluminescence can be powerful to detect and quantify organisms in the ocean. In this study, 17 years of video observations were recorded by remotely operated vehicles during surveys off the California Coast, from the surface down to 3,900 m depth. More than 350,000 observations are classified for their bioluminescence capability based on literature descriptions. The organisms represented 553 phylogenetic concepts (species, genera or families, at the most precise taxonomic level defined from the images), distributed within 13 broader taxonomic categories. The importance of bioluminescent marine taxa is highlighted in the water column, as we showed that 76% of the observed individuals have bioluminescence capability. More than 97% of Cnidarians were bioluminescent, and 9 of the 13 taxonomic categories were found to be bioluminescent dominant. The percentage of bioluminescent animals is remarkably uniform over depth. Moreover, the proportion of bioluminescent and non-bioluminescent animals within taxonomic groups changes with depth for Ctenophora, Scyphozoa, Chaetognatha, and Crustacea. Given these results, bioluminescence has to be considered an important ecological trait from the surface to the deep-sea.

In marine environments, the presence of light plays a major role in the spatial distribution of marine communities. However, in the photic zone during nighttime and in the deeper parts of the water column, where sunlight has been absorbed, animals live in perpetual dim light or darkness^1^. Light emitted by organisms is called bioluminescence; this emission of cold light is due to a biologically generated chemiluminescent reaction. It is an active ability to communicate, in contrast to the passive traits of fluorescence or phosphorescence in which photons are absorbed by a tissue or structure and then re-emitted at a different wavelength. Moreover, bioluminescence is known to play many roles in intra- and inter-specific interactions^2^. Due to the wide diversity of organisms using this process, bioluminescence has also been utilized to detect biological activities in the deep ocean^3^,^4^, the presence of pelagic animals^5^,^6^,^7^,^8^,^9^,^10^,^11^, and to evaluate biomass for oceanographic studies^12^. A robust description of the abundance and distribution of organisms able to emit light and their ecological niches in the water column is needed to accurately perform such surveys. To our knowledge, the most complete catalog of known bioluminescent organisms was compiled by Herring^13^ and updated more recently^2^,^14^. For coastal environments less than 2.5% of the species are estimated to be bioluminescent^15^, while for pelagic environments, this percentage is considerably higher. Indeed, the earliest studies estimate that bioluminescence occurs in approximately 70% of fish species^16^, and by number of individuals, 90% of fishes observed below 500 m depth in the eastern North Atlantic were said to be bioluminescent^16^,^17^. For decapod shrimp (Crustacea) 80% of individuals from the surface to 500 m depth and 41% between 500 and 1,000 m depth were said to be bioluminescent^17^,^18^. Later studies described bioluminescence capability for a range of jellyfish (Cnidaria)^2^,^19^ and established a value of about 90% for species of planktonic siphonophores^20^ and ctenophores^21^. Because of this surprisingly high percentage, these estimates for pelagic species have been intensively used since, in scientific publications^22^,^23^,^24^,^25^ and for outreach on marine biology. However, these studies were limited by: focusing on certain restricted taxonomic categories (siphonophores, ctenophores, jellyfish); being based on general phylogenetic description of bioluminescence ability within taxa; or using non-quantitative approximations. Well documented data sets on diverse taxa are still needed to evaluate the importance of this capability across marine diversity. A primary interest of understanding the distribution of bioluminescent and non-bioluminescent taxa is to examine ecologic niches with co-occurring bioluminescent taxa, and establish the niches shared with taxa where biological interaction or avoidance could be due to light emission. Another interest of collating this information across a large number of taxa is to identify gaps in our understanding of bioluminescence capability for marine ecosystems. Because depth is the main spatial variable driving the distribution of organisms, the development of deep-sea technologies such as cameras, remotely operated vehicles (ROVs), and non-destructive sampling methods has increased the number of dives and *in situ* observations in the pelagic ocean.

This study is based on *in situ* video observations performed by the MBARI ROVs over the last 17 years in the eastern Pacific. Each observation in the videos has been identified taxonomically and its bioluminescence capability has been associated from the literature. Using this information, we quantify the distribution of bioluminescent and non-bioluminescent organisms from the surface to the deep-ocean across 13 taxonomic categories (8 phyla), and 553 phylogenetic concepts (species, genera, families) of organisms observed. Finally, this study shows that bioluminescence is important in the water column, as an ecological trait, spanning the range of depths and the diversity of organisms.

## Results

### Taxonomic observations

**Figure 1:**
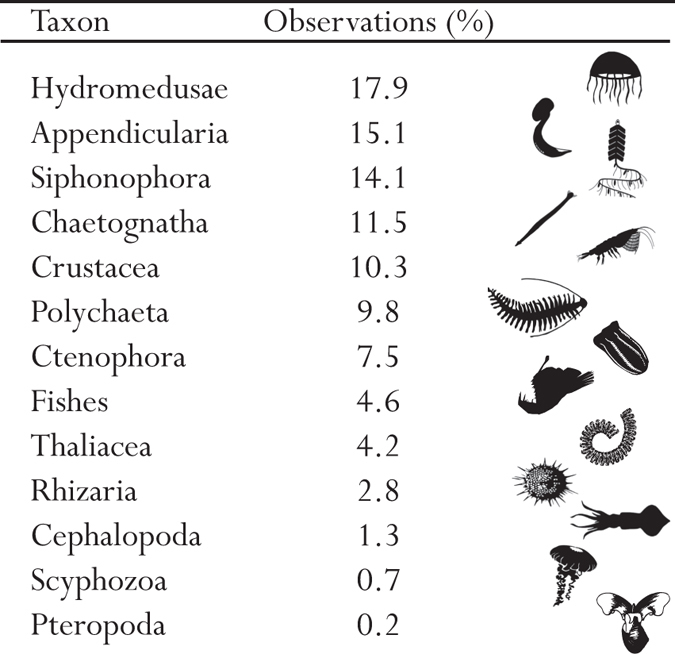
Percentage of observations for each taxon. In this Figure, the monophyletic taxa are Cnidaria (Hydromedusae, Siphonophora, and Scyphozoa) with 32.7% of the data, Mollusca (Pteropoda and Cephalopoda) with 1.5% of the data and, Urochordata (Thaliacea and Appendicularia) with 23.9% of the data. Scyphozoa silhouette from http://phylopic.org, by Mali’o Kodis, photograph by Ching (http://www.flickr.com/photos/36302473@N03/) (license https://creativecommons.org/licenses/by/3.0/). All other are in the public domain, accessible at http://phylopic.org.

A total of 350,536 water-column observations were annotated from videos taken during 240 ROV dives between 1999 and 2016, with about 3/4 of the data between 2006 and 2012. This large amount of data in number, depth, and the large time coverage gives reliable patterns of the vertical distribution of bioluminescence features, which may be considered largely representative of the deep-ocean. The data set, having been gathered during periodic cruises and only during the day, is less suitable for analysis of seasonal or long-term trends, or subtleties like vertical migrations. This data set focuses only on planktonic or pelagic species; any predominantly benthic organisms, such as echinoderms, anthozoans, and ascidians were pre-filtered from the data set, although they can include bioluminescent entities. For analyzing trends, organisms were grouped into broader taxonomic categories, based on functional groupings and broader bioluminescent patterns (see Materials and Methods) section. For example Cnidaria were split into hydromedusae, siphonophores, and scyphozoans because these three groups are readily identifiable and have different patterns with depth. Chordates were sorted into three categories because fishes are functionally very different from urochordates, and within the urochordates, appendicularians are mainly luminous while Thaliacea (salps and doliolids) are mainly non-luminous. Initially organisms were placed into 14 of these taxonomic categories, but the group Nemertea (about 0.1% of the data set), representing 3 concepts, was excluded from further analyses because of its low numbers of observations. The rest of the 553 concepts belonging to 13 broader taxonomic categories ranged from 0.2% (Pteropoda) to 17.9% (Hydromedusae) of the observations (Fig. 1), and these were further analyzed for trends.

Crustacea are pelagic planktonic species mainly represented in our dataset by mysids, decapods, and euphausiids. Infrequently annotated copepods were excluded due to inability to identify them from videos and inconsistencies in how well organisms smaller than a few mm have been annotated. Ctenophores and cnidarians are the most abundant gelatinous organisms. Within cnidarians, Siphonophora, Hydromedusae, and Scyphozoa have been divided into different taxa in this study based on known differentiated behaviors and distributions. Appendicularia are pelagic tunicates producing a feeding structure called a “house”, known to contain bioluminescent inclusions and to be a significant fraction of the organic material sinking to the oceans depths. Fishes mainly represent the marine ray-finned fishes, but for this analysis we have included sharks, while the Pteropoda is a place-holder for pelagic gastropods and also includes heteropods (non-luminous) and pelagic nudibranchs (often luminescent).

### Distribution of bioluminescence over depth

**Figure 2:**
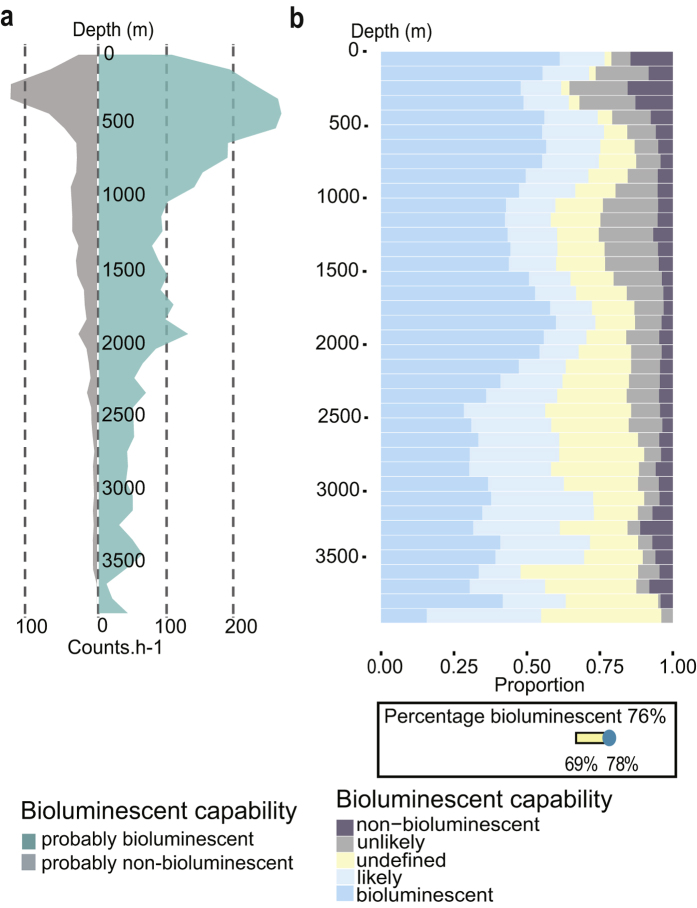
Distribution of bioluminescence capability over depth. (**a**) Number of observations (counts per hour) through the water column for probably non-bioluminescent (non-bioluminescent and unlikely) and probably bioluminescent (bioluminescent and likely) organisms. (**b**) Proportion of bioluminescence capability distributed over depth. In the lower box, the overall percentage of bioluminescent organisms is represented as 76%. The variability of this percentage of bioluminescent capability, depending on the how undefined animals might be assigned, is added on the yellow bar (from 69 to 78%, see text for more details).

The total number of counts per hour is represented (Fig. 2a), for probably bioluminescent and probably non-bioluminescent organisms, after normalization of the data set. The total sum of counts per hour (including bioluminescent, non-bioluminescent from Fig. 2 and undefined, not shown) increased from the surface to the maximum of 411 counts per hour at 350 m depth (bioluminescent and non-bioluminescent, respectively 265 and 131 counts per hour, Fig. 2, and undefined with 15 counts per hour). Then, the number of counts per hour decreased with depth to the lowest value of 14 counts per hour of operation at 3,650 m depth.

Bioluminescent and likely bioluminescent organisms were dominant in the entire water column (in blue, Fig. 2b), ranging between 48 and 77% of the organisms observed. The non-bioluminescent and unlikely bioluminescent organisms represented a small portion of the observations (in dark grey, Fig. 2b), ranging between 2 and 35%. These numbers do not total 100% due to animals undefined for bioluminescence, accounting for between 2 and 43% of the observations. Raw numbers of observations in each depth bin were normalized using the amount of time spent at each depth. As might be expected, the upper part of the water column has a lower percentage of undefined organisms than the less known deeper waters. However, when omitting the undefined organisms, in the global data set, probably bioluminescent organisms accounted for 76% of all observations (down, Fig. 2b), and the probably non-bioluminescent reached 24%. The variability of these percentages over depth is low, and the variability due to undefined organisms is also relatively constant. Indeed, the percentage varies only a small amount, from a low of 69%, if all undefined animals are assumed to be non-luminous, to 78% if they are all assigned as bioluminescent.

### Distribution of bioluminescence within taxa

**Figure 3:**
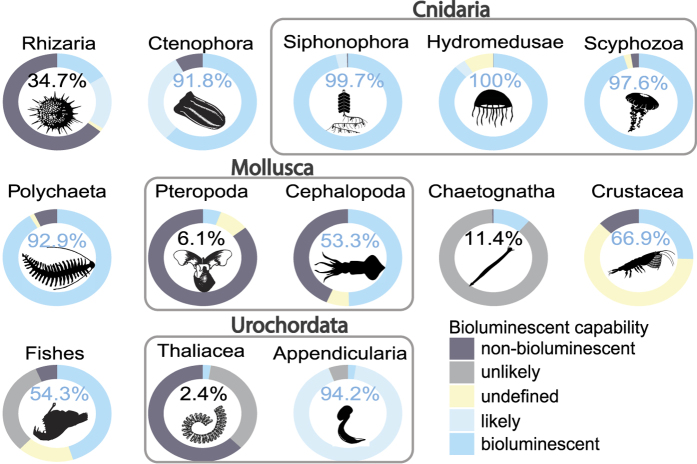
Bioluminescence capability over the main observed taxa. The percentages only represent the probably bioluminescent organisms relative to the sum of probably bioluminescent and probably non-bioluminescent ones. The undefined organisms were not taken into account in these percentages. The color of the typography represents the dominance of the capability. Grey bounding boxes show larger taxonomic groups: Cnidaria (Hydromedusae, Siphonophora and Scyphozoa), Mollusca (Pteropoda and Cephalopoda) and Urochordata (Thaliacea and Appendicularia). Scyphozoa silhouette from http://phylopic.org, by Mali’o Kodis, photograph by Ching (http://www.flickr.com/photos/36302473@N03/) (license https://creativecommons.org/licenses/by/3.0/). All other are in the public domain, accessible at http://phylopic.org.

The proportion of bioluminescent observations calculated for each of the 13 main taxonomic categories showed clear taxon-specific trends (Fig. 3). In this part of the analysis, the undefined observations were not taken into account when computing the percentage of probably bioluminescent observations within each taxon. As with the water-column calculations, these numbers are based on *in situ* observations of organism abundance (total counts), and not on the number of species within each group, meaning that the abundance of some numerically dominant organisms could drive the observed trends. For example, within the Polychaeta, *Poeobius meseres*, which is bioluminescent, was observed in high abundance at all the sampling stations and dives, especially in the deeper waters, and thus was largely responsible for the pattern seen in the Polychaeta. This worm was found with a maximum abundance at about 1,800 m depth. Other bioluminescent-dominant taxa included Appendicularia (94.2% of probably bioluminescent), Polychaeta (92.9%), Ctenophora (91.8%), and all the subgroups of cnidarians *i.e*.: Siphonophora (99.7%), Hydromedusae (100.0%) and Scyphozoa (97.6%) were bioluminescent dominant.

In contrast, Rhizaria (34.7% probably bioluminescent), Chaetognatha (11.4%), Pteropoda (6.1%) and Thaliacea (2.4%) were mainly probably non-bioluminescent with a low diversity of identified luminous species. For Chaetognatha, the recently studied *Caecosagitta macrocephala* and *Eukrohnia fowleri* were the only two bioluminescent species observed^26^. Only two species of bioluminescent Pteropoda (Haddock, pers. obs.) and four species of bioluminescent Thaliacea were also observed (*Doliolula equus, Paradoliopsis harbisoni, Pseudusa bostigrinus* and *Pyrosoma atlanticum*).

Each of the three sub-groups of cnidarians are clearly bioluminescent dominant (no Cubozoa, which are probably all non-bioluminescent, were observed). The two groups of urochordates, Thaliacea and Appendicularia, show completely contrasting dominance for bioluminescence capability (2.4 and 94.2% of probably bioluminescent, respectively). It also has to be noted, for Crustacea and fishes, a substantial fraction of the observations remain undefined, representing together about 15% of the total observations for those groups. We explain this further in the discussion.

### Distribution of bioluminescence over depth and taxa

**Figure 4:**
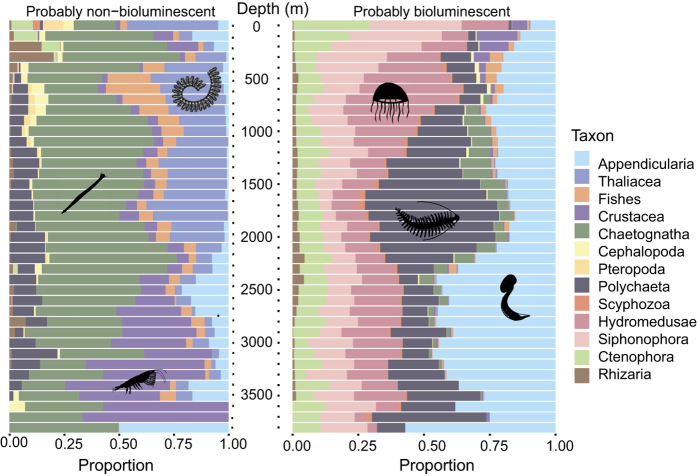
Probably non-bioluminescent and probably bioluminescent taxonomic categories over depth. On the left, taxonomic make-up of the probably non-bioluminescent (including unlikely) observations. On the right, taxonomic components of the probably bioluminescent observations (including likely). The total number of observations differs between the two panels and across depths (see Fig. 2a), but the proportion between 0 and 1 of each group is represented over depth (0 to 3,900 m) using bins of 100 m. Silhouettes are in the public domain, accessible at http://phylopic.org.

The proportion of observations within each taxon shows variability over depth (Fig. 4), taking into account only the probably bioluminescent and probably non-bioluminescent entities.

The photic zone, above 100 m depth, shows a unique distribution of taxa for both probably non-bioluminescent and for probably bioluminescent organisms, compared to deeper depths, which have a more uniform taxonomic make-up. For the probably non-bioluminescent taxa, this shallow layer was mainly composed of Thaliacea, Chaetognatha, Ctenophora, and Pteropoda while for the probably bioluminescent taxa Siphonophora, Ctenophora, and Hydromedusae were dominant. Below 100 m, the probably non-bioluminescent taxa were dominated by Chaetognatha, Thaliacea and Crustacea. Chaetognatha were dominant almost continuously, from 0 to 3,900 m depth, while Thaliacea were mainly present shallower than 2,100 m, and Crustacea below 2,100 m. For the probably bioluminescent taxa, below 100 m, a succession of dominant taxa appeared from the surface to the meso- and bathypelagic zones. Firstly, Siphonophora was the most represented bioluminescent group from the surface to about 500 m. Then, the Hydromedusae dominated the distribution from 500 m to 1,500 m followed by Polychaetes, dominant down to 2,250 m depth. In the deepest layer, below 2,250 m depth, the Appendicularia were the most represented bioluminescent taxon, although they were also well represented throughout the entire water column.

**Figure 5:**
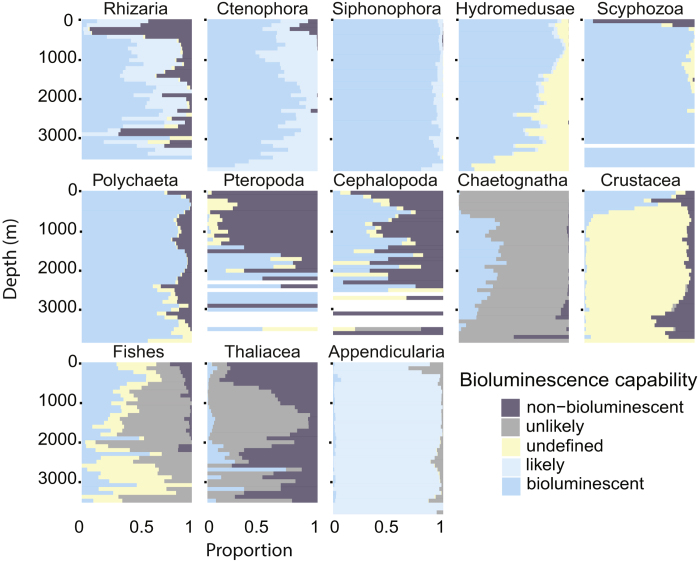
Proportion of the bioluminescence capability over taxonomic categories and depth. The proportion between 0 and 1 of each group is represented using bins of 100 m from 0 to 3,900 m.

**Figure 6:**
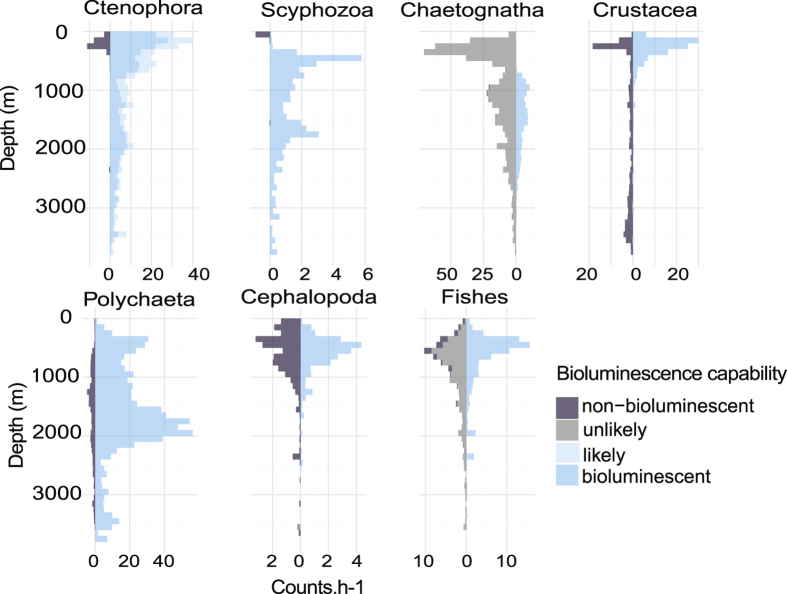
Number of counts per hour over taxonomic categories and depth. The number of counts of animals is normalized per hour for each group and is represented over depth (0 to 3,900 m) using bins of 100 m.

Within each taxonomic category, the proportion of observations belonging to each of the 5 classes of bioluminescence capability was tabulated within depth bins across the full range of depth (Fig. 5). Siphonophora, and Polychaeta had a homogeneous distribution with no clear pattern that varied with depth, mainly due to the fact that more than 99% of them are probably bioluminescent with a low portion of undefined (Fig. 3). In contrast, Ctenophora, Scyphozoa, Pteropoda, Chaetognatha, Crustacea, and Thaliacea show large changes in the distribution of bioluminescence capability through the depths. A clear pattern is observed for Crustacea: bioluminescent Crustacea were mainly observed above 500 m depth (krill, mesopelagic shrimp), while the non-bioluminescent ones (isopods, decapods) were observed below 2,500 m with a gap in between. For Ctenophora, Scyphozoa, and Pteropoda the non-bioluminescent observations predominate in the upper part of the water column (above 500, 200 and 1,500 m respectively). Cephalopoda, Polychaeta, and fishes show the same proportion for probably bioluminescent and non-bioluminescent over depth, with differences in the numbers of counts only (Fig. 6). The probably non-bioluminescent Ctenophora and Scyphozoa (and to some extent Chaetognatha on Fig. 6) are strongly represented in the epipelagic zone and above 500 m but almost absent below. For Ctenophora, this is attributable to the two non-luminous genera (*Hormiphora* and *Pleurobrachia*) being constrained to shallow depths, and for Chaetognatha, the only two luminous species mainly occur below 700 m. For Scyphozoa, *Chrysaora fuscescens* is the main non-bioluminescent species exclusively observed in the upper part of the water column (above 200 m). Figure 6, showing the number of observations over depth, gives a quantification of the distribution patterns. Although the proportion of non-bioluminescent Scyphozoa is high in the upper part of the water column, the absolute numbers of this taxon remain low (Figs 1 and 6). Similarly, the Crustacea show strong proportional patterns, but the absolute counts of deep observations (below 1,000 m) are relatively low.

Finally, it is notable that most of the undefined animals, particularly in Hydromedusae, Polychaeta, and fishes occurred in the deeper parts of the water column (Fig. 5), mirroring the pattern seen in the combined observations (Yellow bars in Fig. 2b). Moreover, because bioluminescent organisms are strongly represented in Hydromedusae and fishes (Fig. 3), it will be of interest to examine deeper representatives of these groups collected in good condition, to discover previously undocumented bioluminescence capabilities.

## Discussion

### ROV detection and associated biases

The use of ROVs fitted with high-definition cameras is a powerful way to conduct observations and surveys over long time scales in the deep-sea^27^,^28^. This method does require a great deal of effort, including time at sea aboard a support ship, video annotation, and organism identification, but it provides an unparalleled view of the abundance and diversity of macroscopic deep-sea organisms. Indeed, ROVs provide a large coverage in space, time, and depth with the ability to investigate the deep sea over a dive lasting several hours^29^. Moreover, the accuracy of the data recorded by video camera is also highly valuable for exploration, in several ways. It allows classification to more precise taxonomic levels than acoustical and other methods, and there is the potential to update annotations over time to keep pace with evolving classifications and species descriptions. The archived images also allow the verification of the data, with recognition of artifactual annotations of organisms (dead or sinking animals, misidentification or identification revised by experts) during the data processing.

One shortcoming is that for a few active species, in particular some fishes, crustaceans and cephalopods, the lights (and sounds) of the vehicle may lead to avoidance behavior, and rarely to attraction^30^,^31^,^32^. Such behaviors are known to be a reaction to bright artificial light, motor noise, electrical fields and vehicle-induced water motion^33^,^34^. In particular, potential reaction to light has to be taken in consideration for bioluminescence studies. The degree to which this can bias quantification is variable and dependent on species. In 2016, Ayma *et al*.^35^, found that fishes would freeze and become motionless in the presence of an ROV, rather than being attracted or fleeing. Some bias is present in our survey, with potentially not all organisms detected by the video cameras. However, the large amount of data collected and the consistency of instruments (3 ROVs and 4 cameras) used over 17 years, and the lack of avoidance response for the majority of organisms analyzed, reinforce the reliability of the survey conducted in this study with the most suitable instrumentation for exploration of the large deep-sea fauna.

When using ROV for camera-based surveys another limitation is the minimum size of organisms that can be recognized. With high-definition video, and depending on the species, this size can be as small as a few millimeters, but typically animals should be larger than a centimeter. This minimum size evolved through the time-span of this study, due to upgrades of the camera sensor and recording at HD resolution. Due to this limit of the ROV for the smallest organisms, this study focused on organisms bigger than one cm. Based on this limitation, copepods (Crustacea) have been removed from the dataset. Indeed, most copepods can only be identified upon close microscopic examination. While this group includes both bioluminescent and non-bioluminescent species, their inclusion would have increased the “undefined’’ group without providing more information on depth-related bioluminescence capability. Ostracods are another group of abundant crustaceans that contain bioluminescent species, but which are too small to be identified using this methodology.

### Bioluminescence description for taxa in the literature

While some estimates of the proportion of bioluminescent organisms in the open ocean have been previously published^16^,^17^,^18^,^27^, to our knowledge, there has been no study based on a thoroughly quantified data set for the full range of midwater taxa. Moreover, the most complete list of bioluminescent taxa in the literature was published 30 years ago^13^ with few addition since^2^,^14^. Because there is a fairly low-level of activity in deep-sea research, and even less work on potentially bioluminescent organisms in good condition, the rate of discoveries of bioluminescent taxa occurs at a very slow decadal rate. Such a slow rate and lack of studies on bioluminescence as an ecological capability is given the high estimates of bioluminescence capabilities (up to 90%) for fauna living in the deep ocean^27^. Our study quantifies that 76% of the organisms observed have the ability to emit bioluminescence. This value is remarkably consistent throughout a 3,900-meter depth range. Although our work is limited to organisms above one cm in size, and may miss some especially reclusive fish, crustacea and cephalopods due to the escape behavior, this value is the most accurate and consistent current estimate across a very broad depth range. Our results also highlight that for some taxa such as Ctenophora and cnidarians (including Siphonophora, Hydromedusae, and Scyphozoa) this percentage was higher than 90%. In contrast, Chaetognatha, Pteropoda, and Thaliacea showed the opposite pattern, with less than 15% of organisms observed being bioluminescent, and most of those newly documented. In fact, until recently, chaetognaths, doliolids and pteropods were considered to be the three main planktonic groups that had no bioluminescent representatives^13^. Based on the prevalence of bioluminescence capability within Hydromedusae, and because about 9% of observations are organisms with undefined capability, it will be interesting to continue exploration of the full extent of luminescence in this group. One of the interesting patterns was that the uppermost layer contained the predominance of non-bioluminescent species of scyphozoan jellyfish. This shallow group includes the most commonly encountered medusae such as *Aurelia* (moon jellies) and *Chrysaora* (sea nettles), which are not bioluminescent. Although they are abundant shallow, they are not a significant portion when considering the water-column as a whole. Deeper, the most abundant scyphozoans are coronate medusae which have been shown to have dramatic bioluminescent displays^36^. It will be very interesting in the future to examine the bioluminescent capabilities of the recently discovered deep Ulmaridae (Scyphozoa), relatives of the moon jellies, such as *Tiburonia*^37^, *Deepstaria*, and *Stellamedusa*^38^.

### Representativeness of the water column

The sampled area is located offshore of central California, and stations extent out into the California current, one of the major coastal currents affiliated with upwelling zones^39^ and blooms^40^. The California Current is part of the North Pacific Gyre, occupying the northern basin of the Pacific. One of the distinctive features of the sampled zone is the distance to the continental shelf break. In this stretch of coast, the shelf is relatively narrow, so that the abyssal seafloor is within a few hundred km from shore. These results, therefore, which are not limited to one particular canyon, should be somewhat representative of other deep-sea waters, comparable to other well-studied areas such as the Porcupine Abyssal Plain station in the North Atlantic (49°N 16.5°W; 4,800 m)^41^.

Our results, interestingly, showed a low variability in the percentages of probably bioluminescent and probably non-bioluminescent organisms over depth. This study is restricted to daytime observations, the dives being almost exclusively conducted between 06:00 and 19:00 local time. Organisms occurring above the oxygen minimum zone (above 700 meters) may undergo day/night vertical migration through the water column. Interestingly, the organisms living in the twilight zone actively use their bioluminescence during day and night. They do not undergo daily changes in their bioluminescent capabilities like surface-dwelling dinoflagellates and crustaceans. Our results, therefore, should be considered to apply to the daytime depths of these shallower deep-sea taxa, while still representative of the deeper and non-migrating taxa. Examining the effects of organism migrations and chronobiological rhythms will be interesting material for future studies.

Several studies measuring bioluminescence intensity using bathyphotometers^42^, or high sensitivity video cameras^43^,^44^,^45^ found a decrease in the bioluminescence intensity recorded with depth. One important implication of our results is that if the proportion of bioluminescent organisms remains stable over depth, as we found, then such decreases are principally related to the decrease of biomass (Fig. 2a). In future works, the relationship between such decrease of abundance and the decrease of bioluminescence measured *in situ* over depth will be interesting to investigate. These future investigations, based on our results, could assess the effectiveness of bulk bioluminescence measurements as a reliable proxy for water-column biomass.

## Conclusions

Bioluminescence is frequently viewed as an exotic phenomenon, but its widespread occurrence and the high diversity of organisms with this capability support that it serves many important ecological roles^2^. Our study found that 76% of oceanic marine organisms observed in deep waters offshore of California have the capability of bioluminescence. This percentage is surprisingly stable throughout the water column, from the surface to the deep sea, although the dominant taxonomic groups contributing to this proportion change over depth. *In situ* measurements of bioluminescence profiles, which decline with depth, are potentially a powerful proxy to detect the changes in biomass with depth and in different water masses. The full extent of bioluminescence capability is yet to be established, especially in the deep sea where continued discoveries await. However, given that the deep ocean being the largest habitat on earth by volume, bioluminescence can certainly be said to be a major ecological trait on earth.

## Materials and Methods

### Sampling using Remotely Operated Vehicles (ROVs)

**Figure 7:**
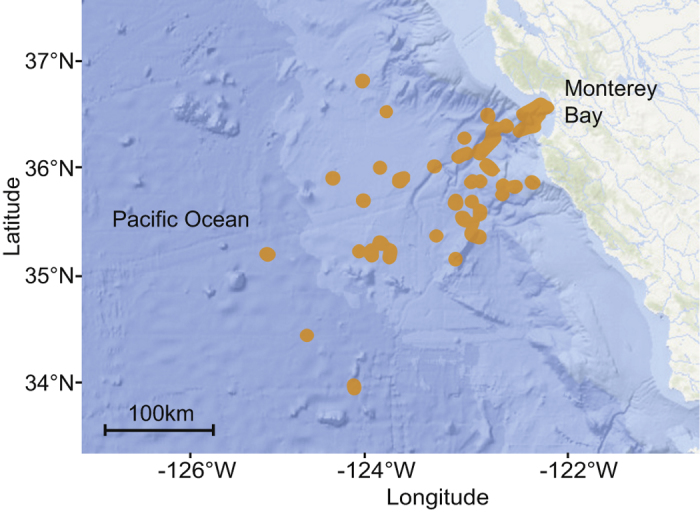
Sampling stations. Map of the eastern Pacific Ocean and California coast showing the sampling stations (orange dots) from March 1999 to June 2016 in the greater Monterey Bay region. The map is based on NOAA bathymetry (https://maps.ngdc.noaa.gov/viewers/bathymetry/), and sampling stations have been represented using R software^47^.

The data were recorded off California, from nearshore waters to 300 km offshore, (latitude from 34.23° to 37.00°N and longitude from 125.02° to 121.73°W) during 240 cruises exploring down to 3,900 m depth (Fig. 7). The study covered the water column within diverse areas from the Monterey canyon to the abyssal plain, and from relatively shallow coastal waters to deep pelagic habitats. During the 17 years sampled, from March 1999 to June 2016, several ROVs were used (Tiburon, Doc Ricketts, Ventana) and 4 cameras were mounted (Panasonic 3-chip, Sony 3-chip, Ikegami HDL40 and Sony HDTV). The focus distance was defined as 1.5 m from the camera. The typical volume observed by the camera varied between 1.2 and 3 m^3^ during this period, although our normalization method is not dependent on this value. The ROV video transects were annotated by staff experts using the Monterey Bay Aquarium Research Institute’s open source Video Annotation Reference System (VARS)^46^ for database entry. Each observation of an animal was logged as a concept, defined at its most specific phylogenetic level observable from video, along with concurrent physical parameters (depth, location). After filtering and quality checks, the final analyzed data set included 350,536 entities within 553 taxonomic concepts (species or higher).

### Data treatment

The observations’ depths were discretized into 100-m bins from 0 to 3,900 m. Because the ROV spends less time at the deepest depths, in order to obtain comparable values over depth independent of the time spent for observation, the data were normalized by the total time spent (in hour) per 100-m depth bin. This study focuses on the water column, so benthic taxa were removed from the observations. Data treatment has been performed using Python scripts for retrieval and normalization, and R version 3.3.1^47^ for stats and plotting.

### Bioluminescence capability attribution

A database of concepts (taxonomic entities) was annotated for the capability of bioluminescence. The capability was classified into one of the following five categories: bioluminescent, likely, undefined, unlikely, and non-bioluminescent, Table 1. Those descriptions are mainly based on previous literature^2^,^13^,^14^ and supplemented with additional unpublished discoveries and observations since (Haddock, pers. obs.). They have been collated for each taxon and are accessible online through the “Deep Sea Guide’’ from MBARI (http://dsg.mbari.org/dsg/home).

For classifying an organism’s capability, as an example, *Aequorea* was defined as bioluminescent^48^. On the opposite end, for the non-bioluminescent category, *Pleurobrachia* was described as non-bioluminescent based on the literature^21^. An example from the undefined category is the Hydromedusa *Ptychogastria* that has never been described for this capability. The categories with the most likely and unlikely observations are Ctenophora (comb jellies), Chaetognatha (arrow worms), and Appendicularia (larvaceans). In the case of Ctenophora, all members examined are luminous except for certain benthic species (not included in this study) and the genera *Pleurobrachia* and *Hormiphora*, which are restricted to fairly shallow waters. The deep-sea ctenophores that could not be identified to a precise taxonomic level are mostly species that have not been given names yet. These are all luminous, so when there are undefined ctenophores from deeper depths it is *likely* that they are luminous species. For chaetognaths, the inverse is true: nearly all are non-luminous except for two orange-colored deep-living species. If a chaetognath therefore was not specifically identified, then it is unlikely to be one of these two distinct luminous species, and therefore it is catalogued as *unlikely*. Non-specific appendicularians observed are mainly small animals, which are most abundantly in the luminous genus *Oikopleura*, and they are visible due to the presence of their mucus house, which acts as a particle accumulator. For these there is a *likely* probability that the observed and non-described appendicularians are bioluminescent.

In this work and several of the subsequent plots, the bioluminescent and likely-bioluminescent were grouped into “probably bioluminescent’’ and the non-bioluminescent and unlikely-bioluminescent were grouped into “probably non-bioluminescent’’. Data sets and the script (Rmarkdown under R-Studio) of the representations are available as Supplementary Information (S1 to S3 datasets).

## Additional Information

**How to cite this article:** Martini, S. and Haddock, S. H. D. Quantification of bioluminescence from the surface to the deep sea demonstrates its predominance as an ecological trait. *Sci. Rep.* **7**, 45750; doi: 10.1038/srep45750 (2017).

**Publisher's note:** Springer Nature remains neutral with regard to jurisdictional claims in published maps and institutional affiliations.

## Supplementary Material

Supplementary Data

## Figures and Tables

**Table 1:** Bioluminescence classification.

Bioluminescent	Taxon described with bioluminescence capability in the literature.
Likely	Taxon probably bioluminescent based on taxonomic assignment and observations.
Non-bioluminescent	Taxon described as non-bioluminescent in the literature.
Unlikely	Taxon probably non-bioluminescent based on taxonomy and observations.
Undefined	Includes two cases: organisms that could not be assessed because they were equally likely to be bioluminescent or not, as well as organisms whose bioluminescent capability has not been examined or reported.
